# The Effectiveness of a Mobile National Remote Emergency System for Malignant Hyperthermia in China: Retrospective Pre-Post Implementation Study

**DOI:** 10.2196/71476

**Published:** 2025-08-14

**Authors:** Lingcan Tan, Hong Yu, Yunxia Zuo, Bin Liu, Tao Zhu, Xiaoqian Deng

**Affiliations:** 1 Department of Anesthesiology, West China Hospital, Sichuan University, No. 37 Guoxue Alley, Wuhou District, Chengdu, 610041, China, 86 02885423593

**Keywords:** malignant hyperthermia, rare disease, mobile healthcare, WeChat, dantrolene, outcome, anesthesia, emergency, pre-post study

## Abstract

**Background:**

Malignant hyperthermia (MH) seriously threatens perioperative safety. Historically, limited awareness of MH among anesthesiologists and the unavailability of dantrolene have caused a high mortality rate of MH events in China. Although domestic dantrolene has been available in China since 2020, Chinese anesthesiologists continue to face significant challenges in managing MH crises. A WeChat applet–based National Remote Emergency System for Malignant Hyperthermia (MH-NRES) was developed to assist anesthesiologists in making rapid diagnosis, initiating dantrolene mobilization, implementing effective treatment, and subsequently constructing an MH database. However, the effectiveness of MH-NRES in real-world patients experiencing MH in China remains uncertain.

**Objective:**

This study aimed to assess the effectiveness of MH-NRES in enhancing outcomes for patients with MH.

**Methods:**

A retrospective pre-post implementation study was conducted from January 2018 to November 2024. The MH-NRES intervention was initiated in December 2022. Medical records were reviewed both before and after the implementation of our intervention, encompassing demographic characteristics, anesthesia-related data, treatment details, and clinical outcomes. Descriptive analyses and a pre-post intervention comparison were used to assess the effectiveness of the MH-NRES intervention. The primary outcome was the mortality of patients with MH. The use of dantrolene and the time interval from the MH episode to the administration of dantrolene were considered secondary outcomes. The user activity metrics of MH-NRES were also reported.

**Results:**

After the MH-NRES was launched for public use, the cumulative number of users reached 21,835, with a maximum daily user growth of 689 (median 15, IQR 9‐25). The cumulative page views amounted to 245,740 and the average daily page views were 262.8. A total of 34 patients with MH and 14 patients with MH were retrospectively collected before and after the intervention, respectively. The mortality of patients with MH in the postimplementation group was significantly lower compared with that in the preimplementation group (1/14, 7.1% vs 19/34, 55.9%; *P*=.002). No significant differences were observed in the early clinical manifestations of MH between the 2 groups. The rate of dantrolene use in the postimplementation group was significantly higher than that in the preimplementation group (11/14, 78.6% vs 15/34, 44.1%; *P*=.03). The dantrolene available time in the postimplementation group was 126.5 minutes earlier than that in the preimplementation group, but the difference did not reach statistical significance (median 113.5, IQR 54.5‐244.3 vs 240, IQR 105-324 minutes, *P*=.08).

**Conclusions:**

The MH-NRES aids in improving the timely administration of dantrolene and decreasing mortality rates among patients with MH. This system represents a rare disease perioperative management model and constitutes a specialized perioperative management approach for rare diseases that suits the current medical situation in China.

## Introduction

Malignant hyperthermia (MH) is a rare autosomal dominant genetic disorder triggered by certain anesthetics, which threatens the perioperative safety of patients with high mortality [1,2]. Early recognition and prompt treatment of the MH crisis are essential for a successful rescue [3]. Once MH is highly suspected, the most critical treatment is to administer IV dantrolene as early as possible [4,5]. Nevertheless, substantial systemic challenges hinder effective MH emergency response in China. Current evidence indicates that Chinese anesthesiologists frequently lack comprehensive MH knowledge, clinical experience, and standardized training protocols [6,7]. In addition, the stock of dantrolene in Chinese hospitals at present is far from optimal even after it is commercially available in China after 2020 [6]. Inexperience and lack of dantrolene contribute to a significantly higher mortality rate of MH in China compared with Europe and the United States [8-10].

To address these systemic gaps, innovative solutions leveraging digital health technologies have emerged as promising intervention strategies. Mobile health, which is defined as health monitoring and information exchange through smartphones and wireless devices, demonstrates the potential for overcoming geographical barriers, supply limitations, and time-sensitive consultation needs [11-15]. This technological paradigm aligns with the demonstrated demand among patients with rare diseases for digital platforms providing both medical guidance and psychological support [16-18]. Building upon this foundation, the China Malignant Hyperthermia Emergency Assistance (CMHEA) group was founded in 2015, essentially a chat group integrated within China’s largest social application, WeChat. This pioneering mobile health intervention created an unprecedented communication channel enabling: (1) real-time professional consultation during MH crises, (2) coordinated dantrolene mobilization across medical institutions, and (3) knowledge dissemination through case-based learning. Over the past 5 years, the CMHEA has facilitated remote MH crisis interventions and successfully rescued patients [9].

However, the utilization rate of dantrolene remains significantly below the international standard, resulting in a persistently high mortality rate associated with MH [10]. Therefore, the CMHEA group may fall short in addressing the prevalent challenges associated with MH first aid. Currently, the traditional chat group assistance model faces the following issues: first, many anesthesiologists are unaware of the existence of the CMHEA group, resulting in a lack of a communication platform to facilitate connections between anesthesiologists in need of assistance and those available for remote consultation; second, the capacity of the WeChat group is limited, which may be insufficient to support the growing number of primary hospitals seeking remote assistance for MH emergencies; third, during emergencies, the influx of numerous and unorganized messages within chat groups hinders efficient assistance. The rapid flow of unprioritized information complicates the timely retrieval of crucial data, leading to wasted time on information screening and reduced rescue efficiency. These operational challenges highlight the urgent need for an optimized MH emergency response system that preserves CMHEA’s proven strengths while addressing its structural limitations.

Given the current shortage within the CMHEA group, we have designed a mobile-based National Remote Emergency System for Malignant Hyperthermia (MH-NRES) [19]. The system is a WeChat mini program or applet, which enables users to conduct searches directly within the WeChat platform, eliminating the need to download an additional application. Its ease of accessibility and simplicity of operation make it highly suitable for use in emergency situations. Chinese anesthesiologists are encouraged to use the system to acquire knowledge and skills related to MH. In addition, the system could assist anesthesiologists in making rapid diagnoses, initiating dantrolene mobilization from other hospitals in China, executing effective treatment, and subsequently constructing an MH database [19,20] (Figure S1 in Multimedia Appendix 1). The MH-NRES has been tested to be valid and reliable, and the assessors found it straightforward to acquire professional knowledge, enhance their confidence during MH rescue, and demonstrate a favorable attitude towards the utilization of the system [21]. There has been a case report supporting the use of MH-NRES, which can improve anesthesiologists’ ability to manage intraoperative MH [22].

Given the absence of real-world analysis among patients with MH, we conducted a retrospective pre-post implementation study. In this study, our objectives were as follows: (1) to evaluate the effectiveness of the MH-NRES in improving the survival rates of patients with MH and (2) to assess the MH-NRES’s impact on enhancing the accessibility of dantrolene.

## Methods

### Study Design and Participants

This study was a retrospective pre-post implementation clinical trial conducted from January 2018 to November 2024. The MH-NRES intervention was initiated in December 2022 to assist anesthesiologists in managing the MH crisis. The clinical diagnosis of MH was confirmed based on the guidelines provided by the Association of Anesthetists for emergency settings. These criteria included (1) an unexplained, unexpected increase in end-tidal carbon dioxide (P_ET_CO_2_); (2) an unexplained, unexpected increase in heart rate; (3) an unexplained, unexpected increase in temperature; and (4) a positive response to dantrolene administration [5]. The MH Clinical Grading Scale (CGS) was used to qualitatively assess the probability of the MH cases. Patients were included if their MH-CGS score exceeded 35 points (CGS grades 5‐6). Our study adopted a lower threshold (CGS≥35) for 3 reasons. First, the clinical manifestation of MH is highly variable, while typical MH cases constitute a limited proportion of total cases. Second, the CGS score is dynamic and may increase or fluctuate with disease progression, so including patients with scores ≥35 ensures the early detection of subtle symptoms. Finally, previous studies have used a CGS score of ≥35 as an inclusion criterion, validating this threshold [9,10]. Given the lack of in vivo contracture test (IVCT) capabilities in China, genetic testing was performed postcrisis whenever feasible. Cases with incomplete or missing data also were excluded.

### Interventions

Before the implementation of MH-NRES (January 2018 to December 2022), anesthesiologists addressed MH events either by relying on their clinical experience or by seeking assistance from the CMHEA WeChat group for support. Experts within this group would provide remote diagnosis and treatment guidance. Additionally, dantrolene was primarily obtained through direct communication with nearby hospitals.

After the implementation of MH-NRES (December 2022 to November 2024), when a patient with suspected MH presents, the anesthesiologists could use the MH-NRES, which facilitates rapid web-based diagnosis, provides treatment measures, and offers guidance on the use of dantrolene. Additionally, the system provides a link to the CMHEA group for further assistance. The anesthesiologists also have the option to initiate dantrolene mobilization, with the system automatically prioritizing available resources based on dantrolene inventory levels and geographical proximity, thereby furnishing contact details for hospitals that can administer dantrolene.

### Data Collection

All data were collected through the MH database in MH-NRES. In this study, data collection was divided into 2 phases: preimplementation and postimplementation of the MH-NRES. During the postimplementation phase, anesthesiologists meticulously documented and completed the standard case report forms (CRFs) following the treatment of patients with MH. Prior to the implementation of the MH-NRES, the absence of a standardized nationwide platform necessitated the adoption of retrospective data collection methods. Specifically, our research team collaborated closely with the anesthesiologists in charge of treatment through the CMHEA group. Detailed guidance was provided regarding the study objectives and the requirements for completing the CRFs. Additionally, researchers actively assisted the anesthesiologists in recalling and documenting clinical information as comprehensively as possible, thereby ensuring the completion of CRFs consistent with the postimplementation version. Despite the inherent limitations of retrospective data collection, the authenticity and reliability of the data were maximized through rigorous collaboration and communication between the research team and the treating anesthesiologists.

Demographic data (age, gender, body weight), family MH history, previous anesthetic experiences with unusual metabolic responses, and type of surgery were collected. Anesthesia data included the type of anesthetics, the stage of MH onset (whether it occurred during anesthesia induction, intraoperatively, or postoperatively), the location of MH onset (such as operating room, postanesthesia care unit, ICU, etc), the time intervals from anesthetic induction to the documentation of the first sign of MH symptoms, such as rapidly increasing temperature, elevated P_ET_CO_2_ levels, masseter spasm, generalized muscular rigidity, tachycardia, hypercarbia, hypertension, and others. Additionally, the maximal records of temperature, serum potassium, P_ET_CO_2_, arterial blood carbon dioxide (PaCO_2_), base excess, creatine kinase, myoglobin, coagulation function parameters, renal function parameters, and the CGS score were also recorded. The treatment process, including the dantrolene available time (time interval from MH episode to the administration of dantrolene) and the rate of dantrolene use, need for blood purification, and mortality were collected. For user activity metrics of MH-NRES, we collected the cumulative number of users, daily user growth, daily visitors, and daily page views. Additionally, the number of visits to each module of the MH-NRES was reported.

The primary outcome was the mortality of patients with MH. The use of dantrolene, the dantrolene available time, and the use of hemopurification were considered secondary outcomes.

### Statistical Analyses

Data analyses were performed using SPSS Statistics (version 27.0; IBM). All continuous variables were initially assessed for normality. In this study, the Shapiro-Wilk test was used for normality due to the small sample size. Then we presented the mean and SD for continuous variables with normal distribution and the median and IQR for continuous variables with skewed distributions. The categorical variables were presented as numerals and percentages. Statistical comparisons between the groups were conducted using 2-tailed independent *t* test or Mann-Whitney *U* test (for mean and median, respectively) for continuous variables. For categorical variables, comparisons are performed using chi-square or Fisher exact test. A 2-sided *P* value <.05 was considered statistically significant.

### Ethical Considerations

This study was carried out in accordance with the ethical standards of the Helsinki Declaration (sixth revision) and was approved by the Ethical Committee on Clinical Research of West China Hospital of Sichuan University (approval number: 2022‐875; October 27, 2022). All data were collected and processed anonymously to protect personal privacy. Informed consent was sought from patients or their relatives before their data were collected in the database. Due to the nature of the retrospective study, we did not provide compensation to the participants. The trial was registered in the Chinese Clinical Trial Registry (ChiCTR2200066497; December 7, 2022).

## Results

### Promotion of the MH-NERS Following Its Deployment

After the MH-NRES launched for public use in May 2022, the cumulative number of users reached 21,835, with a maximum daily user growth of 689 (median 15, IQR 9‐25). The maximum number of daily visitors was 767, with a median of 27 (IQR 18‐39). The cumulative page views amounted to 245,740, and the average daily page views were 262.8. The average visit duration was 141.2 seconds. The module that users visit most often was the MH treatment (13,768 of 49,465, 27.8%), followed by the dantrolene mobilization (13,547 of 49,465, 27.4%), then the instruction on dantrolene use (7029 of 49,467, 14.2%) (Table 1). By December 2024, the MH-NRES had incorporated a total of 89 institutions holding dantrolene reserves, with this number steadily increasing annually. These 89 institutions are distributed across 28 cities in 16 provinces, autonomous regions, or municipalities throughout mainland China.

**Table 1.** Distribution of visiting in each module.

Module	Users visiting, n (%)
Treatment of MH^a^ crisis	13,768 (27.8)
Dantrolene mobilization	13,547 (27.4)
Instruction on dantrolene use	7029 (14.2)
Simulation training	4929 (10.0)
Quick diagnosis	4302 (8.7)
MH guidelines	3878 (7.8)
Post MH crisis	2012 (4.1)

a MH: malignant hyperthermia

### Baseline Characteristics of Included Patients With MH

**Figure 1.**
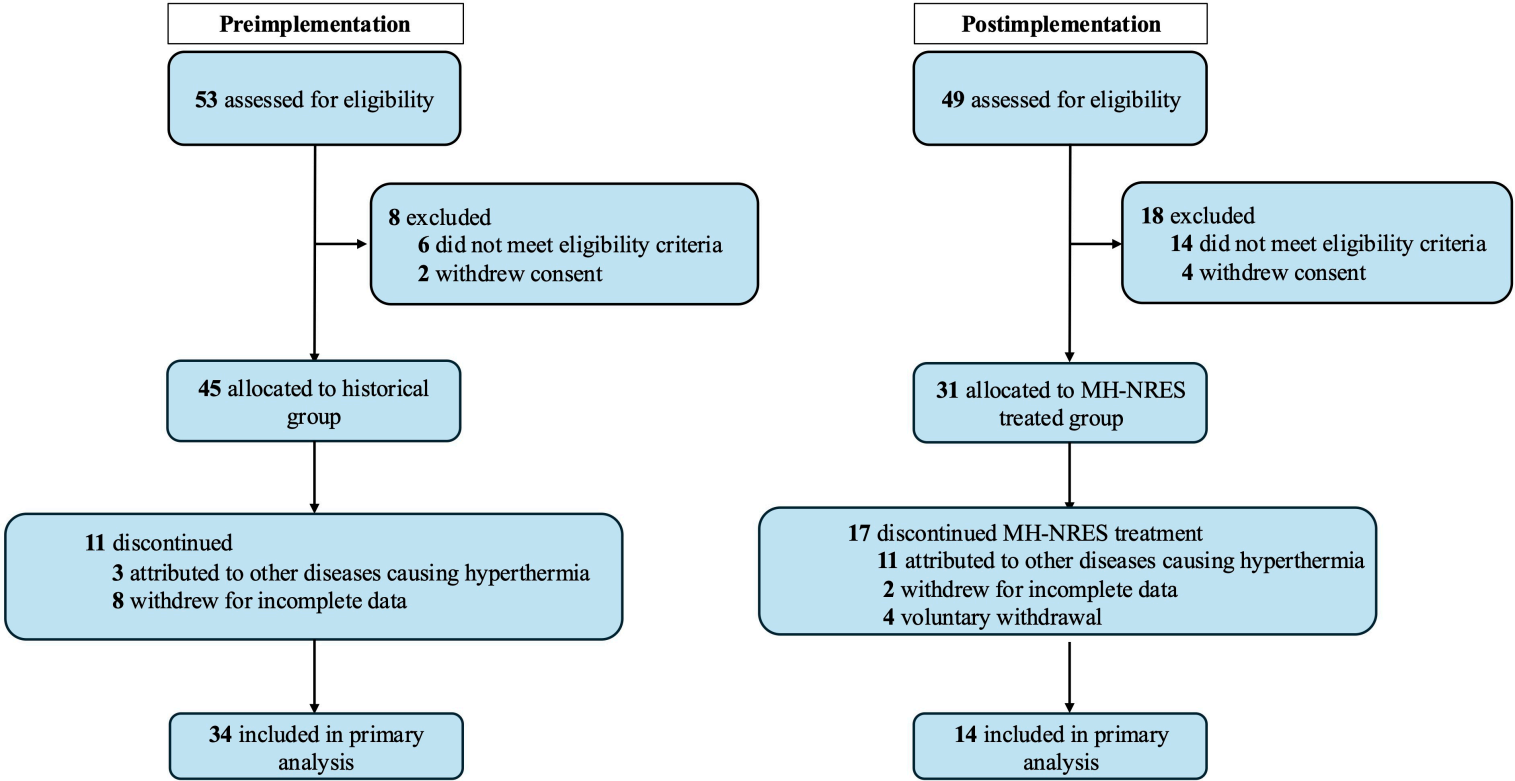
Flow diagram. MH-NRES: National Remote Emergency System for Malignant Hyperthermia.

**Figure 2.**
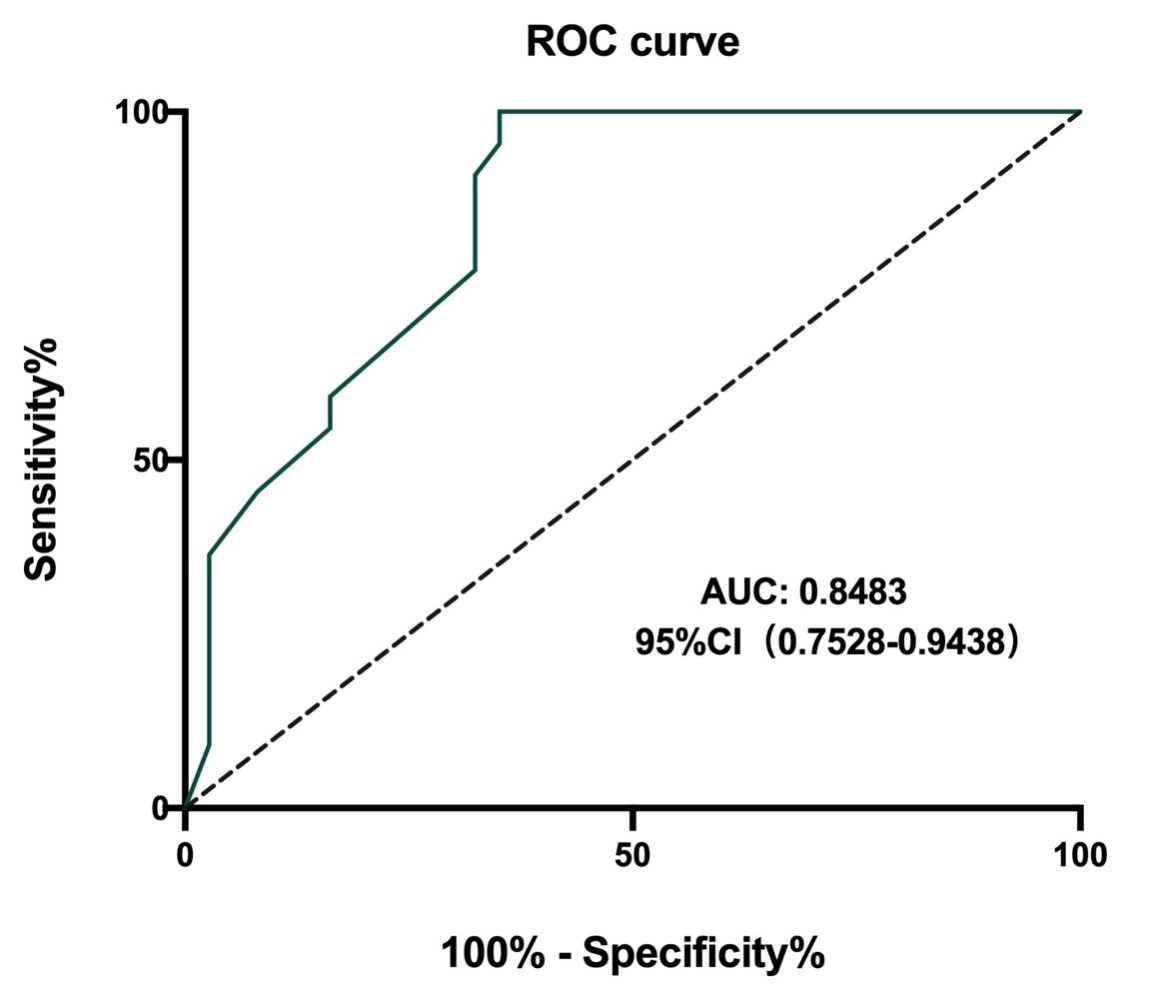
ROC curve of CGS score for diagnosing MH. AUC: area under the receiver operating characteristic curve; CGS: Clinical Grading Scale; MH: malignant hyperthermia; ROC: receiver operating characteristic.

A total of 48 patients were analyzed during the study period, including 34 patients in the preimplementation phase and 14 patients in the postimplementation phase. The workflow of this study is shown in Figure 1. In the preimplementation group, 53 patients presenting with ambiguous signs of MH were identified as potential cases. Of these, 8 individuals were excluded due to high fever caused by infection or excessive thermal retention. Additionally, 11 withdrew from the study due to incomplete data, resulting in a final enrollment of 34 participants. In the postimplementation group, we received 49 urgent requests for assistance from various hospitals via the MH-NRES. Six patients with CGS scores below 20 (somewhat less than likely) were excluded, while 8 patients with CGS scores ranging from 20 to 34 (somewhat greater than likely) were ultimately ruled out after undergoing repeated assessments and expert consultations. Among the remaining patients with CGS scores above 35, 14 patients were included following a comprehensive evaluation that encompassed clinical manifestations, auxiliary examination results, and expert consultations, which culminated in a definitive clinical diagnosis of MH (Table 2). Subsequent receiver operating characteristic curve analysis for CGS score revealed that a cut-off point of 45.5 provides the best clinical diagnosis for MH (sensitivity of 100% and specificity of 64.9%). The area under the receiver operating characteristic curve was 0.848 (95% CI 0.753‐0.944) (Figure 2).

Among the 48 patients with MH, 83.3% (n=40) patients were male. The median age was 38 (IQR 12‐40.5) years, with 39.6% (n=19) patients of 2‐17 years, 45.8% (n=22) patients of 18‐50 years, and 14.6% (n=7) patients older than 50 years. Only one patient had a family MH history; none of the patients had previous anesthetic experiences with unusual metabolic responses. Regarding the use of triggering anesthetics, most patients (47/48, 97.9%) were administered only volatile anesthetics, specifically sevoflurane (45/48, 93.8%), desflurane (1/48, 2.1%), and isoflurane (1/48, 2.1%). Only one case (1/48, 2.1%) used both succinylcholine and volatile anesthetics (desflurane). The 48 patients in this study involved 10 surgery types. No differences were observed in baseline characteristics between the 2 groups (Table 3).

**Table 2.** Clinical characteristics of patients with malignant hyperthermia (MH) managed by the National Remote Emergency System for Malignant Hyperthermia (n=14).

Case	1	2	3	4	5	6	7	8	9	10	11	12	13	14
Sex	F^a^	F	M^b^	F	M	M	M	M	M	M	M	M	F	M
Age (years)	9	9	13	53	24	57	10	16	37	24	25	40	34	54
Volatile agents	Sevo^c^	Sevo	Sevo	Sevo	Sevo	Sevo	Sevo	Sevo	Sevo	Sevo	Sevo	Sevo	Sevo	Des^d^
Succinylcholine	N^e^	N	N	N	N	N	N	N	N	N	N	N	N	N
MH onset time, min	75	5	60	30	43	158	70	5	150	20	76	120	25	60
The first CGS^f^	38	33	48	25	48	38	30	43	35	48	38	30	38	48
The second CGS	53	48	63	50	65	43	53	53	45	58	48	58	46	53
The third CGS	78	53	73	65	—^g^	63	63	58	48	75	—	63	—	61
Initiation of therapy (min)	3	10	15	20	20	4	15	3	5	2	5	3	5	2
Dantrolene mobilization	Y^h^	Y	Y	N	Y	Y	Y	Y	Y	N	Y	Y	N	Y
Successful mobilization	N	Y	Y	—	N	Y	Y	Y	N	—	Y	Y	—	Y
Use of dantrolene	N	Y	Y	Y	N	Y	Y	Y	N	Y	Y	Y	Y	Y
Dantrolene available time (min)	—	235	239	62	—	107	418	120	—	134	32	260	5	90
Survival	Y	Y	Y	Y	N	Y	Y	Y	Y	Y	Y	Y	Y	Y

a F: Female.

b M: Male.

c Sevo: Sevoflurane.

d Des: Desflurane.

e N: No.

f CGS: Clinical Grading Scale.

g Not available.

h Y: Yes.

**Table 3.** Baseline characteristics of the patients before and after initiation of national malignant hyperthermia (MH) remote emergency system intervention in patients with MH.

Characteristics	Preimplementation (n=34)	Postimplementation (n=14)	*P* value
Age (years), n (%)			.70
≤17	14 (41.2)	5 (35.7)	
18-50	16 (47.1)	6 (42.9)	
≥50	4 (11.8)	3 (21.4)	
Male, n (%)	30 (88.2)	10 (71.4)	.21
Family MH history, n (%)	1 (2.9)	0 (0)	.50
Hospital type, n (%)			.57
Public	32 (94.1)	12 (85.7)	
Private	2 (5.9)	2 (14.3)	
Hospital grades, n (%)			>.99
Grade-A Tertiary	31 (91.2)	13 (92.9)	
Grade-B Tertiary	3 (8.8)	1 (7.1)	
Triggering anesthetics, n (%)			>.99
Volatile +, Succinylcholine –	33 (97.1)	14 (100)	
Volatile +, Succinylcholine +	1 (2.9)	0 (0)	
Volatile –, Succinylcholine +	0 (0)	0 (0)	
Surgery type, n (%)			.29
General surgery	7 (20.6)	7 (50)	
Otolaryngology	3 (8.8)	1 (7.1)	
Cardiovascular	1 (2.9)	1 (7.1)	
Urological	4 (11.8)	1 (7.1)	
Orthopedic	5 (14.7)	1 (7.1)	
Plastic	4 (11.8)	1 (7.1)	
Oral	1(2.9)	1 (7.1)	
Gynecological	0 (0)	1 (7.1)	
Ophthalmologic	5 (14.7)	0 (0)	
Neurosurgery	4 (11.8)	0 (0)	

### Clinical Manifestations of Patients With MH Between the 2 Groups

The mean time from exposure to the triggering agents until the onset of MH clinical manifestations was significantly shorter in the postimplementation group compared with the preimplementation group (71.5, SD 50.2 vs 143.4, SD 104.4 minutes; *P*=.04). The incidence of early clinical signs of MH, including rapid elevation in body temperature, abnormal increase in P_ET_CO_2_, muscle rigidity, and sinus tachycardia, did not demonstrate statistically significant differences between the 2 groups. The incidence of hyperkalemia was significantly lower in the postimplementation group than in the preimplementation group (6/14, 42.9% vs 28/34, 82.4%; *P*=.01), while no statistically significant differences were observed regarding acute renal failure and disseminated intravascular coagulation between both groups, as detailed in Table 4.

**Table 4.** Comparison of clinical manifestations between 2 groups of patients.

Clinical manifestations	Preimplementation (n=34)	Postimplementation (n=14)	*P* value
Early clinical signs			
MH^a^ onset time (min), mean (SD)	143.4 (104.4)	71.5 (50.2)	.04
Rapidly increasing temperature, n (%)	29 (85.3)	13 (92.9)	.66
Elevation of P_ET_CO_2_^b^, n (%)	28 (82.4)	14 (100)	.16
Muscular rigidity, n (%)	19 (55.9)	11 (78.6)	.14
Sinus tachycardia, n (%)	27 (79.4)	14 (100)	.09
Advanced clinical manifestations			
Hyperkalemia, n (%)	28 (82.4)	6 (42.9)	.01
Acute renal failure, n (%)	10 (29.4)	3 (21.4)	.73
DIC^c^, n (%)	11 (32.4)	2 (14.3)	.29
Laboratory results			
Max Temperature (℃), mean (SD)	41.6 (1.6)	40.5 (2.0)	.16
Max P_ET_CO_2_ (mmHg), mean (SD)	114.8 (37.4)	111.4 (37.6)	.83
Max PaCO_2_^d^ (mmHg), mean (SD)	93.6 (19.4)	86.3 (26.2)	.47
Max BE^e^ (mmol/L), mean (SD)	–12.3 (5.5)	–6.3 (6.5)	.03
Max K^f^ (mmol/L), mean (SD)	6.6 (1.0)	5.5 (1.0)	.02
Max CK^g^ (U/L), median (IQR)	9574 (4374‐20322)	3177 (1370.5‐13753.5)	.50
Max MYO^h^ (ng/mL), mean (SD)	3683.9 (2614.6)	2599.15 (2865.5)	.36
CGS^i^ score, mean (SD)	52.8 (11.6)	60.7 (11.0)	.04

a MH: malignant hyperthermia.

b P_ET_CO_2_: end-tidal carbon dioxide partial pressure.

c DIC: disseminated intravascular coagulation.

d PaCO_2_: arterial partial pressure of carbon dioxide.

e BE: base excess.

f K: potassium.

g CK: creatine kinase.

h MYO: myohemoglobin.

i CGS: Clinical Grading Scale.

There were no significant differences in peak body temperature, peak P_ET_CO_2_, peak PaCO_2_, peak CK, and peak myoglobin between the 2 groups (*P*>.05). However, in comparison to the preimplementation group, patients in the postimplementation group demonstrated significantly lower peak serum potassium levels (5.5, SD 1.0 vs 6.6, SD 1.0 mmol/L; *P*=.02) and peak BE concentration (−6.3, SD 6.5 vs −12.3, SD 5.5; *P*=.03). The CGS score of patients in the postimplementation group was higher (60.7, SD 11 vs 52.8, SD 11.6; *P*=.04), as shown in Table 4.

### Outcomes Comparison of Patients With MH Between the 2 Groups

#### Overview

The mortality of patients with MH in the postimplementation group was significantly lower than that in the preimplementation group (1/14, 7.1% vs 19/34, 55.9%; *P*=.002). More patients in the postimplementation group received dantrolene administration than those in the preimplementation group (11/14, 78.6% vs 15/34, 44.1%; *P*=.03). The interval from the MH episode to the administration of dantrolene was 126.5 minutes shorter in the postimplementation group compared with the preimplementation group; however, no statistically significant difference was observed between the 2 groups (median 113.5, IQR 54.5‐244.3 vs 240, IQR 105-324 minutes, *P*=.08). Four patients (11.8%) in the preimplementation group and 3 patients (21.4%) in the postimplementation group received continuous blood purification, with no significant difference (*P*=.40) (Table 5).

**Table 5.** Comparison of emergency treatment and outcomes between 2 groups of patients.

Variables	Preimplementation (n=34)	Postimplementation (n=14)	*P* value
Use of dantrolene, n (%)	15 (44.1)	11 (78.6)	.03
Dantrolene available time (min), median (IQR)	240 (105‐324)	113.5 (54.5‐244.3)	.08
Use of hemopurification, n (%)	4 (11.8)	3 (21.4)	.40
Mortality, n (%)	19 (55.9)	1 (7.1)	.002

During a median follow-up period of 28 months (up to March 2025), 12 of 13 patients (92.3%) in the postimplementation group achieved favorable functional recovery. Three patients experienced major complications requiring specialized interventions:

#### Neurological Sequelae

A 13-year-old male patient (case 3) received continuous renal replacement therapy for 7 days due to acute renal failure, followed by transient muscle weakness and speech dysfunction. After referral to the pediatric rehabilitation center, the patient achieved complete recovery of motor and language function following a systematic neurological rehabilitation intervention.

#### Thromboembolic Event

A 16-year-old male patient (case 8) developed basilic vein thrombosis following a 48-hour dantrolene infusion administered to prevent the recurrence of MH. Thrombus resolution was successfully achieved within 10 days through treatment with enoxaparin (4000 IU, every 12 hours) for 4 days, followed by oral rivaroxaban therapy. The patient was discharged on the 14th postoperative day, with no residual deficits.

#### Late Mortality

A 53-year-old female patient (case 4) was admitted to the hospital with massive gastrointestinal bleeding 24 months after cervical cancer surgery. She first underwent jejunal artery embolization under monitored anesthesia care and then an emergency laparotomy under total intravenous anesthesia (propofol-remifentanil regimen). No signs of intraoperative MH were observed, but she died from refractory hemorrhagic shock and multiple organ failure.

## Discussion

Due to the prevalence of digital mobile devices, the potential role of mobile-enabled apps in facilitating health management has been widely investigated, but only a few of them were designed for rare diseases [23-27]. Despite the low attention and small number of potential users, the development of mobile applications related to rare diseases should not be ignored. To our knowledge, this is the first study reporting that using an applet can have a positive effect on the emergency recovery of patients with MH. This study is aimed at building a mobile health-based remote emergency management platform for MH, thereby solving the difficulties of Chinese anesthesiologists during the MH crisis.

Early identification of MH is essential for reducing MH mortality [28-30]. However, the diversity and atypical clinical manifestations of MH increase the challenges to early diagnosis. While the caffeine-halothane test is recognized as the standard diagnostic method for MH, its implementation has not been widespread in China. Currently, CGS scoring criteria for MH is the most frequently used tool for clinical diagnosis of MH [31,32]. In this study, with the assistance of artificial intelligence analysis, we developed an MH early diagnosis model based on CGS. Leveraging the convenience and speed of a mobile app platform, this model is capable of quickly screening for MH risks, calculating scores using multidimensional data, and providing different warning prompts based on the score level. Furthermore, the system supports dynamic assessment as the patient’s condition evolves, aiding anesthesiologists in conducting rapid clinical diagnoses and implementing effective emergency management strategies. Currently, there is a lack of relevant studies examining the diagnostic efficacy of the CGS score for MH. Our study found that a CGS score cut-off point of 45.5 demonstrated high predictive value for identifying MH, with a sensitivity of 100% and specificity of 64.9%. These results indicated that the CGS score manifested considerable diagnostic value for rapid identification of MH, albeit with its high sensitivity accompanied by a relatively low specificity. Additional evidence-based medical research is requisite to confirm its diagnostic effectiveness. Furthermore, these findings accentuated that even with intelligent assistance, the clinical diagnosis of MH should be complemented by consultations from experienced experts.

The data from this study demonstrated that the onset time in the postimplementation group was shorter in comparison with the preimplementation group. Notably, the CGS score for patients in the postimplementation group was higher, with 10 patients surpassing a CGS score of 35 at the initial assessment. As disease progression occurred, 13 patients recorded a second CGS score exceeding 45, further substantiating the clinical diagnosis of MH. Additionally, the incidence of hyperkalemia was lower in the postimplementation group, and peak serum potassium concentrations were also decreased. These findings suggested that the MH-NRES enhances clinicians’ awareness regarding MH monitoring, subsequently improving their diagnostic capabilities and potentially shortening the duration of clinical diagnosis, while also facilitating the prompt initiation of treatment, which are important measures to secure patient survival.

Given China’s vast territory, large population, uneven distribution of medical resources across regions, and notable disparities in medical standards, this study incorporated social forces, including medical institutions engaged in drug preparation, pharmaceutical enterprises, and distribution agencies, to establish a nationwide dantrolene mobilization network. The results demonstrated that under this novel emergency model, the administration rate of dantrolene has soared to 78.6%. Additionally, the time taken to make dantrolene available time was shortened, ultimately leading to a decrease in the mortality rate of patients with MH. These findings suggest that the mobile MH-NRES effectively establishes a connection between those seeking antidotes and specific drug storage locations, facilitating faster access to essential medications, alleviating challenges such as poor availability, unclear information, and delayed treatments for patients with MH, thereby saving crucial time in dantrolene mobilization. In addition, MH-NRES also provides standardized emergency treatment guidance for MH, reducing the likelihood of errors and omissions and enhancing the standardization and consistency of MH crisis management. Consequently, leveraging the availability of domestic dantrolene reserves, the MH-NRES contributed to lower mortality rates and minimized wastage due to drug expiration, thereby optimizing the utilization of drug resources.

Regarding the time to dantrolene administration, which was shorter in the postimplementation group than in the preimplementation group, no significant difference was observed between the 2 groups. One potential factor that might have influenced the results was the insufficient sample size. Nevertheless, it is noteworthy that the time to dantrolene administration for patients with MH in China is significantly longer than that observed in developed countries [28,32]. Even in the postimplementation group, the median available time for dantrolene remained at 113.5 minutes. In contrast, a previous study reported a median time interval of 45 minutes from the first sign of MH to dantrolene administration in surviving patients [32]. This delay might be closely associated with the limited number of medical institutions equipped with dantrolene, as well as their uneven distribution across China. While some provincial capitals, such as Chengdu and Beijing, have multiple drug storage points to serve their surrounding areas, many others lack such facilities. Consequently, during an MH crisis, dantrolene must be urgently sourced from neighboring provinces and cities, resulting in prolonged waiting times that significantly impact patient outcomes. Despite this, by capitalizing on the steady increase in domestic dantrolene reserves and implementing the MH-NRES, we observed a reduction in mortality among patients with MH, which adds robustness to the study. Starting in 2023, the shelf life of domestic dantrolene injections has been extended to 2 years; this change is anticipated to reduce storage costs and enhance the willingness of major medical institutions to stock this critical drug, thereby improving its accessibility during MH emergencies.

This study has several limitations. First, the diagnosis of MH was based on clinical criteria rather than the gold-standard IVCT, owing to the limited availability of MH diagnostic laboratories in China. Although this approach aligns with international emergency protocols [4,5], it may introduce potential diagnostic uncertainty. The implementation of the MH-NRES underscores the necessity for concurrent development of MH diagnostic infrastructure. Establishing regional MH diagnostic centers equipped with IVCT and genetic testing capabilities in China could address this gap. This centralized model would align with China’s tiered health care system, not only enhancing diagnostic accuracy but also promoting genetic counseling and research collaborations, as demonstrated by the successful model established by the North American Malignant Hyperthermia Registry. Second, given the low incidence of MH, the number of patients enrolled in the study over a 2-year period was relatively small. Consequently, our findings only summarized the preliminary application results of this new emergency model. Future research will necessitate additional time, increased participation from anesthesiologists, and a more comprehensive analysis of treatment outcomes for patients with MH to fully evaluate the efficacy of this emergency approach. Third, the MH database contained a limited number of cases and presented missing data in case reports, such as insufficient detailed information on symptomatic and supportive treatments as well as patient follow-up, which impeded meaningful comparisons between the 2 groups. Finally, a potential bias in patient inclusion was identified in this study, as certain MH cases were not included as they did not use the MH-NRES or contact the CMHEA group. This may affect the generalizability and accuracy of the findings.

In conclusion, the MH-NRES aids in enhancing the clinical diagnostic sensitivity of MH and the timely administration of dantrolene, decreasing mortality rates among patients with MH. This system represents a rare disease perioperative management model and constitutes a specialized perioperative management approach for rare diseases that suits the current medical situation in China.

## Supplementary material

10.2196/71476 Multimedia Appendix 1 The home page of MH-NRES. MH-NRES: National Remote Emergency System for Malignant Hyperthermia.

## Abbreviations

CGS: Clinical Grading Scale
CMHEA: China Malignant Hyperthermia Emergency Assistance
CRF: case report form
IVCT: in vivo contracture test
MH: malignant hyperthermia
MH-NRES: National Remote Emergency System for Malignant Hyperthermia

## References

[1] Rosenberg H, Pollock N, Schiemann A, Bulger T, Stowell K (2015). Malignant hyperthermia: a review. Orphanet J Rare Dis.

[2] Litman RS, Smith VI, Larach MG (2019). Consensus statement of the malignant hyperthermia association of the United States on unresolved clinical questions concerning the management of patients with malignant hyperthermia. Anesth Analg.

[3] Han Y, Qu Y, Wang X The 100 most cited articles in malignant hyperthermia. Anesthesiology and Perioperative Science.

[4] Rüffert H, Bastian B, Bendixen D (2021). Consensus guidelines on perioperative management of malignant hyperthermia suspected or susceptible patients from the European Malignant Hyperthermia Group. Br J Anaesth.

[5] Hopkins PM, Girard T, Dalay S (2021). Malignant hyperthermia 2020: guideline from the association of anaesthetists. Anaesthesia.

[6] Tan L, Yu H, Yan J (2023). The knowledge profile, competence and pending problems of Chinese anesthesiologists in dealing with malignant hyperthermia: a cross-sectional survey. J Multidiscip Healthc.

[7] Wang J, Yu Y, Gao Y (2024). The anesthesiologists’ perception of malignant hyperthermia and availability of dantrolene in China: a cross-sectional survey. Risk Manag Healthc Policy.

[8] Gong X (2021). Malignant hyperthermia when dantrolene is not readily available. BMC Anesthesiol.

[9] Tan L, Teng Y, Yu H (2022). Clinical features of suspected malignant hyperthermia in China from 2015 to 2020: a retrospective study from China malignant hyperthermia emergency assistance group. J Multidiscip Healthc.

[10] Larach MG, Klumpner TT, Brandom BW (2019). Succinylcholine use and dantrolene availability for malignant hyperthermia treatment: database analyses and systematic review. Anesthesiology.

[11] Jaensson M, Dahlberg K, Eriksson M, Nilsson U (2017). Evaluation of postoperative recovery in day surgery patients using a mobile phone application: a multicentre randomized trial. Br J Anaesth.

[12] Marty AP, Braun J, Schick C, Zalunardo MP, Spahn DR, Breckwoldt J (2022). A mobile application to facilitate implementation of programmatic assessment in anaesthesia training. Br J Anaesth.

[13] Plummer K, Adina J, Mitchell AE (2024). Digital health interventions for postoperative recovery in children: a systematic review. Br J Anaesth.

[14] Hao J, Yang L, Wang Y (2023). Mobile prenatal education and its impact on reducing adverse pregnancy outcomes: retrospective real-world study. JMIR Mhealth Uhealth.

[15] Su B, Chen Y, Shen X (2023). Effectiveness of the lilly connected care program in improving glycemic management among patients with type 2 diabetes in china: retrospective real-world study. J Med Internet Res.

[16] Moore J, Goodson N, Wicks P, Reites J (2022). What role can decentralized trial designs play to improve rare disease studies?. Orphanet J Rare Dis.

[17] Yabumoto M, Miller E, Rao A, Tabor HK, Ormond KE, Halley MC (2022). Perspectives of rare disease social media group participants on engaging with genetic counselors: mixed methods study. J Med Internet Res.

[18] Byambasuren O, Beller E, Hoffmann T, Glasziou P (2020). mHealth app prescription in Australian general practice: pre-post study. JMIR Mhealth Uhealth.

[19] Yu H, Tan L, Teng Y (2022). The first national remote emergency system for malignant hyperthermia (MH-NRES) in China: protocol for the design, development, and evaluation of a WeChat applet. JMIR Res Protoc.

[20] Yu H, Tan L, Deng X (2024). Improving dantrolene mobilization in regions with limited availability. Anesthesiology.

[21] Yu H, Tan L, Zhu T, Deng X (2023). A WeChat applet-based national remote emergency system for malignant hyperthermia in China: a usability study. BMC Med Inform Decis Mak.

[22] Xiao Y, Yu R, Gu J (2024). Diagnosis and rescue of malignant hyperthermia induced by anesthesia during radical surgery in a cervical cancer patient using the national remote emergency system: a case report. Medicine (Baltimore).

[23] Hatem S, Long JC, Best S, Fehlberg Z, Nic Giolla Easpaig B, Braithwaite J (2022). Mobile apps for people with rare diseases: review and quality assessment using mobile app rating scale. J Med Internet Res.

[24] Long JC, Best S, Nic Giolla Easpaig B (2022). Needs of people with rare diseases that can be supported by electronic resources: a scoping review. BMJ Open.

[25] Rüther DF, Sebode M, Lohse AW (2021). Mobile app requirements for patients with RARE LIVER diseases: a single center survey for the ERN RARE-LIVER‬‬‬. Clin Res Hepatol Gastroenterol.

[26] Esteban-Vasallo MD, Domínguez-Berjón MF, García-Riolobos C (2019). Effect of mobile phone text messaging for improving the uptake of influenza vaccination in patients with rare diseases. Vaccine (Auckl).

[27] Araújo-Vilar D, Fernández-Pombo A, Rodríguez-Carnero G (2020). LipoDDx: a mobile application for identification of rare lipodystrophy syndromes. Orphanet J Rare Dis.

[28] Larach MG, Brandom BW, Allen GC, Gronert GA, Lehman EB (2014). Malignant hyperthermia deaths related to inadequate temperature monitoring, 2007-2012: a report from the North American malignant hyperthermia registry of the malignant hyperthermia association of the United States. Anesth Analg.

[29] Lu Z, Rosenberg H, Li G (2017). Prevalence of malignant hyperthermia diagnosis in hospital discharge records in California, Florida, New York, and Wisconsin. J Clin Anesth.

[30] Urman RD, Rajan N, Belani K, Gayer S, Joshi GP (2019). Malignant hyperthermia–susceptible adult patient and ambulatory surgery center: society for ambulatory anesthesia and ambulatory surgical care committee of the American Society of Anesthesiologists position statement. Anesth Analg.

[31] Klingler W, Heiderich S, Girard T (2014). Functional and genetic characterization of clinical malignant hyperthermia crises: a multi-centre study. Orphanet J Rare Dis.

[32] Toyota Y, Kondo T, Shorin D (2023). Rapid dantrolene administration with body temperature monitoring is associated with decreased mortality in Japanese malignant hyperthermia events. Biomed Res Int.

