# Supplementary figures and images for: The Effectiveness of a Mobile National Remote Emergency System for Malignant Hyperthermia in China: Retrospective Pre-Post Implementation Study
Source: J Med Internet Res. 2025 Aug 14;27:e71476. doi: 10.2196/71476 (PMC12352588; doi:10.2196/71476)

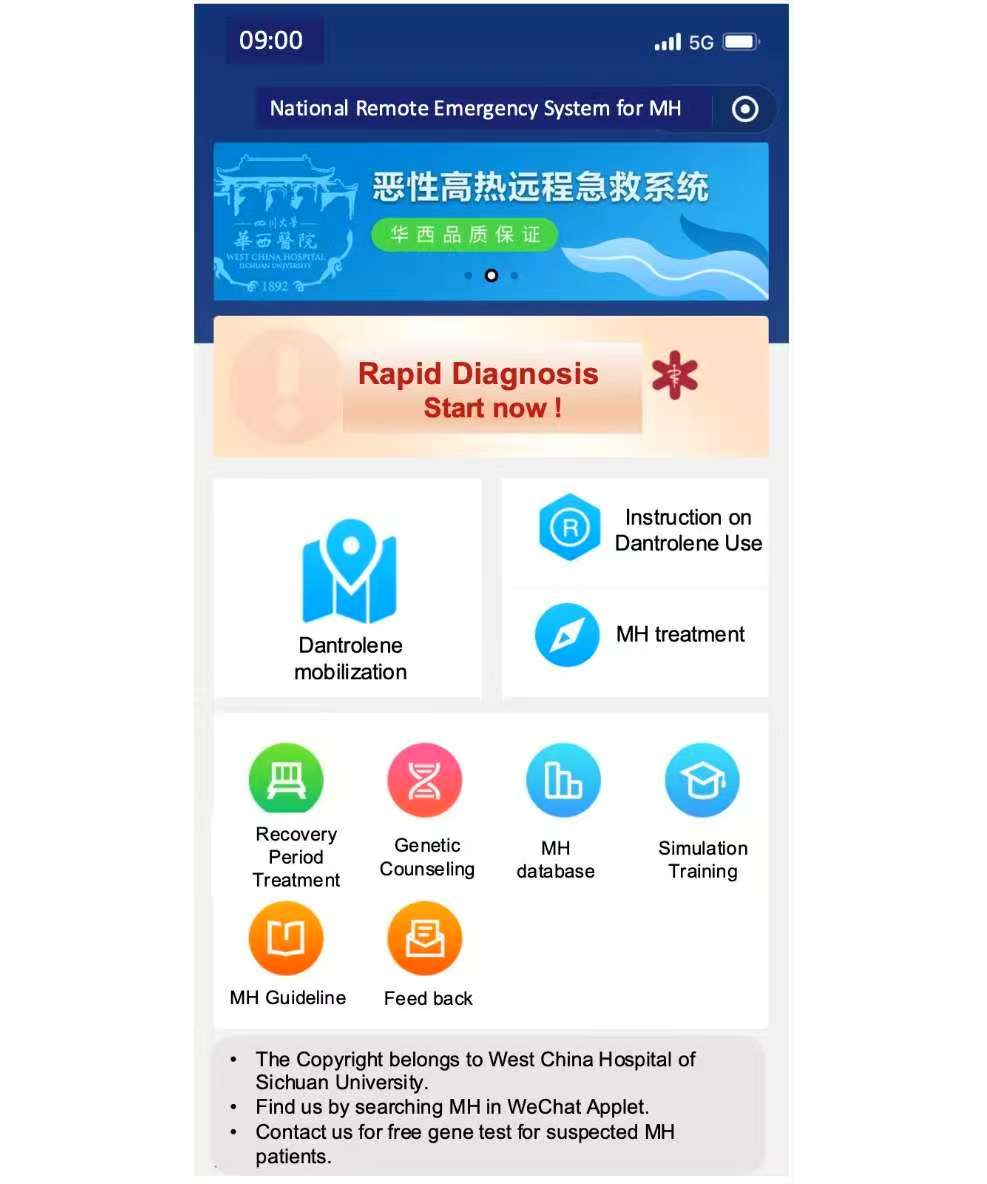

Supplement: Multimedia Appendix 1 [file jmir-v27-e71476-s001.png]
